# Estimating risk propagation between interacting firms on inter-firm complex network

**DOI:** 10.1371/journal.pone.0185712

**Published:** 2017-10-03

**Authors:** Hayato Goto, Hideki Takayasu, Misako Takayasu

**Affiliations:** 1 Department of Computational Intelligence and Systems Science, Interdisciplinary Graduate School of Science and Engineering, Tokyo Institute of Technology, 4259, Nagatsuta-cho, Yokohama 226-8502, Japan; 2 Institute of Innovative Research, Tokyo Institute of Technology, 4259, Nagatsuta-cho, Yokohama 226-8502, Japan; 3 Sony Computer Science Laboratories, 3-14-13 Higashigotanda, Shinagawa-ku, Tokyo 141-0022, Japan; University of Warwick, UNITED KINGDOM

## Abstract

We derive a stochastic function of risk propagation empirically from comprehensive data of chain-reaction bankruptcy events in Japan from 2006 to 2015 over 5,000 pairs of firms. The probability is formulated by firm interaction between the pair of firms; it is proportional to the product of *α*-th power of the size of the first bankrupt firm and *β*-th power of that of the chain-reaction bankrupt firm. We confirm that *α* is positive and *β* is negative throughout the observing period, meaning that the probability of cascading failure is higher between a larger first bankrupt firm and smaller trading firm. We additionally introduce a numerical model simulating the whole ecosystem of firms and show that the interaction kernel is a key factor to express complexities of spreading bankruptcy risks on real ecosystems.

## Introduction

It has been generally recognized that social and economic networks can be captured as complex systems whose nodes are individual agents that interact [1, 2]. Indeed, not only economists but also physicists have shown interest in the complexity underlying the systems and how they work [3–10]. Their contributions provide profound new insights into such areas of interest as cascading failures and resilience [11–20] using the lens of the science of complex systems [21–24]. These theoretical and empirical analyses deepen our fundamental insight into the systems’ dynamics to better predict the impact of economic or social crises, such as subprime mortgages in 2008 [2].

**Fig 1:**
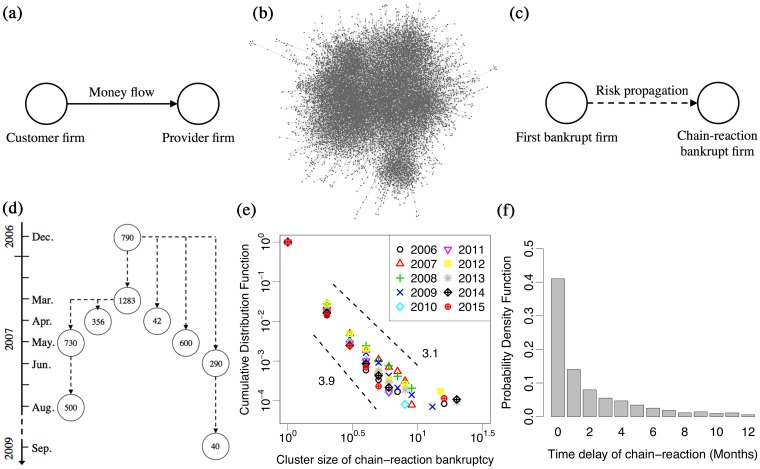
**(a)** A component of the inter-firm business transaction networks. The nodes are firms and links are business transactions from customers to providers, indicating a money flow. **(b)** An example of the inter-firm business transaction network within Kyoto prefecture in 2015. The nodes are firms in Kyoto prefecture and links are business transactions between the firms. The network has 18,391 nodes and 38,540 links and has typical complex network properties such as scale-free property and small world property. **(c)** A component of a cluster of chain-reaction bankruptcies. The cluster nodes are firms and links are risk propagations from first bankruptcies to chain-reaction bankruptcies. **(d)** A nine-size cluster of chain-reaction bankruptcies. Each circle and dotted arrow shows a bankrupt firm and the direction of risk propagation, respectively. The center number is annual sales (million yen) of a bankrupt firm. The top circle shows the first bankrupt firm and the other circles show chain-reaction bankruptcy firms. **(e)** Cluster size distribution of chain-reaction bankruptcies from 2006 to 2015 in log-log scale. As shown in **(d)**, the size of the cluster is defined as the number of bankrupt firms of a cascade reaction. **(f)** Time delay distribution from first bankruptcies to next cascades. About 40% of these events happened within a month and about 85% were within a year.

Similar to other complex networks, business firm networks are complex ecosystems interacting with others in various ways. An inter-firm business transaction network is a typical example. This network, whose nodes are firms that link through business transactions from customers and providers, producing a money flow (opposite from a goods/service flow) as shown in Fig 1(a), has statistical complex properties [25], such as small world property [26] and scale-free property [27], accompanied by various scaling relations [28–34]. Fig 1(b) shows an example of an inter-firm business transaction network within Kyoto prefecture in 2015.

A representative cascading failure of a firm or financial ecosystem is a chain-reaction bankruptcy [35–43], which is defined as bankruptcy triggered by a proceeding default of a firm that trades or has other relations with the bankrupting firm [44]. For instance, Fujiwara et al.[39] conducted a study using a combination of data on a Japanese inter-firm transaction network and a bankruptcy list in 2006. They choose firms randomly from the business network with a probability of bankruptcy; however, the cluster size distribution of chain-reaction bankruptcies, where the size of the cluster is defined as the number of cascade-reaction bankrupt firms, as shown in Fig 1(c), 1(d) and 1(e), was not reproduced by the model. This indicated the additional effect of spreading bankruptcy risks on the real ecosystem. Bardoscia et al.[42, 43] also investigated the effect as shock propagation on bank lending networks. They formulated a microscopic theory by iterating balance sheets and found that the network effects could amplify exogenous shocks in some conditions. As reported in those analyses, risk is spread on an economy based on the complex interplay between agents.

In this study, we analyze comprehensive data of chain-reaction bankruptcy events in Japan from 2006 to 2015 and empirically derive a stochastic function of risk propagation. The probability is formulated by firm interaction between a first bankruptcy firm and a chain-reaction bankruptcy firm. The amount of risk propagation between firms is approximately proportional to the product of *α*-th power of the size of a customer firm and *β*-th power of that of a provider firm, which is very similar to the form of “money flow” from customers and providers [45]. This also indicates that each *α* and *β* of the interaction kernel is positive and negative, respectively. To further our understanding, we introduce a simulation-based network evolution model [46, 47] using inter-firm business transaction networks from 2006 to 2015. The results numerically confirm that the interaction kernel works to reproduce the cluster size distribution of chain-reaction bankruptcies in Japan.

## Materials and methods

### Empirical data analysis

#### Chain-reaction bankruptcy

Business practices in Japan are unique. When building trustworthy relationships or managing credit risk, the Japanese first tend to gather their business partners’ detailed corporate information. Then, professional third-party organizations are used to evaluate their partners’ credit status. Teikoku Databank Ltd (TDB) is one of the largest corporate research providers in Japan; it has been assessing the credit status of firms for 117 years. Their credit research reports include detailed information of the financial statements of firms, their history, business partners, management, and banking transactions. TDB also has detailed information about bankruptcies in Japan. For example, it includes bad debt, which is the total amount of accounts receivable that will not be collected, as a scale of bankruptcy. In this study, we analyze a Japanese firm database collected by TDB that includes chain-reaction bankruptcies.

The list of chain-reaction bankruptcies in the database includes 5,245 pairs of 3,370 first bankruptcy firms and 5,245 chain-reaction bankruptcy firms, hereafter called *f* and *c*, respectively. According to the chain-reaction bankruptcy data from 2006 to 2015 in Table 1, there were 11,644 bankruptcies and 525 chain-reaction bankruptcies per year on average, respectively. There were over 10 chain-reaction firm clusters each year and a little difference in tail (Table 1). In addition, Fig 1(f) shows the distributions of a time delay from first bankruptcies *f* to next cascades *c*. About 40% of these events happened within a month and about 85% were within a year.

**Table 1:** Frequency of bankruptcies and chain-reaction bankruptcies per year from 2006 to 2015 in Japan. There are about 1% bankruptcies of the total number of firms and about 4% chain-reaction bankruptcies of the total number of bankruptcies per year on average.

Year	Number of firms	Number of bankruptcy firms	Number of chain-reaction bankruptcy firms	Maximum cluster size of chain-reaction bankruptcies
2006	1,003,366	11,832	313	16
2007	1,004,275	12,610	785	9
2008	1,017,459	14,346	892	9
2009	1,035,113	13,973	782	13
2010	1,073,941	12,216	526	8
2011	1,114,309	11,788	494	6
2012	1,119,895	11,443	415	15
2013	1,124,316	10,499	383	20
2014	1,131,821	9,168	366	20
2015	1,136,179	8,560	289	16
Ave.	1,076,067	11,644	525	13

#### Scaling relations

**Fig 2:**
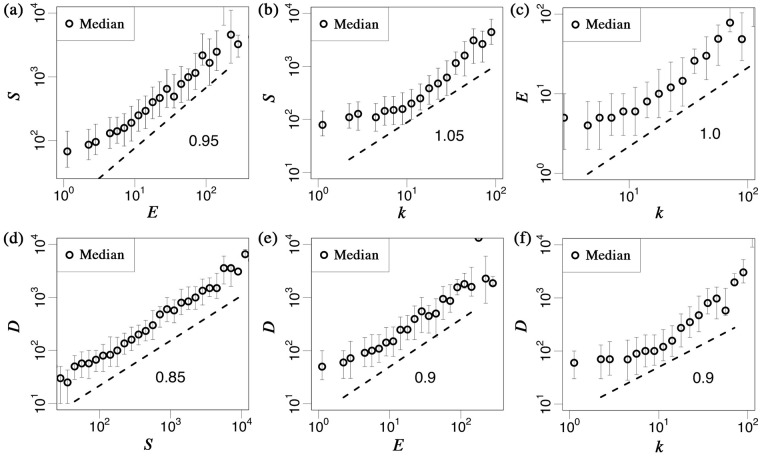
Scaling laws of median of bankrupt firms between pairs of (a) *S* and *E*, (b) *S* and *k*, (c) *E* and *k*, (d) *D* and *S*, (e) *D* and *E*, and (f) *D* and *k* in 2015 in log-log scale, respectively. There are scaling laws of continuing firms such that *S* ∝ *E*^1.3^, *S* ∝ *k*^1.3^, and *E* ∝ *k*^1.0^, however, those of bankrupt firms are different than are observed as *S* ∝ *E*^0.9∼1.0^, *S* ∝ *k*^1.0∼1.1^, and *E* ∝ *k*^1.0∼1.1^. We note that these exponents are observed by the least squares method with coefficient of determination *R*^2^ ≥ 0.9 and the error-bars show first and third quartile, respectively.

In earlier studies on national business networks and their transactions, it was found that there are scaling laws of continuing firms between the median of pairs of sales *S*, number of employees *E*, and number of business transactions *k* such that such that *S* ∝ *E*^1.3^, *S* ∝ *k*^1.3^, and *E* ∝ *k*^1.0^ [33]. The TDB-supplied Japanese firm database also includes a bankruptcy list with each firm’s *S*, *E*, *k*, and bad debt *D*. Fig 2(a), 2(b) and 2(c) show the scaling relations of bankrupt firms between the median of the pairs of *S*, *E*, and *k* in 2015 in log-log scale that are observed as *S* ∝ *E*^0.9∼1.0^, *S* ∝ *k*^1.0∼1.1^, and *E* ∝ *k*^1.0∼1.1^, respectively. The scaling relations of bankrupt firms are different from that of continuing firms, the relation between employees *E* and the business transactions *k* is nearly the same, while sales *S* is nearly a linear function of *E* and *k*. Moreover, Fig 2(d), 2(e) and 2(f) show the scaling relations of bankrupt firms between the median of pairs of *S*, *E*, *k*, and *D* in log-log scale that are observed for *D* ∝ *S*^0.8∼0.9^, *D* ∝ *E*^0.8∼0.9^, and *D* ∝ *k*^0.7∼0.9^, respectively. The larger the firm, the larger its amount of bad debt, nearly proportionally. In the following discussion, we apply *k* as the measure of firm size (it can be substituted by *E* or *S*, given that there are scaling relations among those quantities as described above).

#### Kernel of chain-reaction bankruptcies

**Fig 3:**
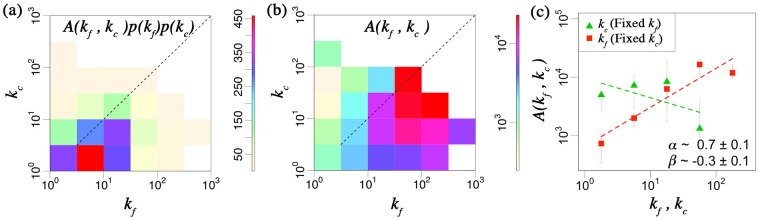
**(a)** Frequency of chain-reaction bankruptcies *A*(*k*_*f*_, *k*_*c*_)*p*(*k*_*f*_)*p*(*k*_*c*_) from 2006 to 2015, where *A*(*k*_*f*_, *k*_*c*_) shows the interaction kernel between the number of business transactions for the first bankrupted firm *k*_*f*_ and that of successive firm *k*_*c*_ and *p* shows the probability density. First bankruptcy firms tend to be larger than their chain-reaction bankruptcy firms. **(b)** Probability of chain-reaction bankruptcies *A*(*k*_*f*_, *k*_*c*_) from 2006 to 2015. The probability of chain-reaction bankruptcies between larger firms is higher than between smaller ones. **(c)** *A*(*k*_*f*_, *k*_*c*_)’s scaling laws with respect to *k*_*f*_ and *k*_*c*_, respectively. Green triangles and red squares show the relation between the median of *A*(*k*_*f*_, *k*_*c*_) and *k*_*c*_ or *k*_*f*_ with fixed *k*_*f*_ or *k*_*c*_ from 2006 to 2015 in log-log scale, respectively. Each dashed-line shows a slope of *α* (red) and *β* (green). The probability of cascading failure is higher between larger first bankruptcy firms and smaller trading firms. We note that these exponents are observed by the least squares method with coefficient of determination *R*^2^ ≥ 0.9 and the error-bars show first and third quartile, respectively.

We observe frequency of bankruptcies as a function of link numbers *k*_*f*_ and *k*_*c*_, the number of business transactions for the first bankrupted firm and that of the successive firm, respectively. For quantity measurement of firms, we focus on the number of business transactions *k*, although it can be substituted as *E* or *S* due to the scaling laws between them as mentioned previously. Fig 3(a) and 3(b) show the distributions of frequency of chain bankruptcy *A*(*k*_*f*_, *k*_*c*_)*p*(*k*_*f*_)*p*(*k*_*c*_) and the interaction kernel *A*(*k*_*f*_, *k*_*c*_), where *p*(*k*_*f*_) and *p*(*k*_*c*_) denote the probability densities of *k*_*f*_ and *k*_*c*_, respectively. We note that *A*(*k*_*f*_, *k*_*c*_)*p*(*k*_*f*_)*p*(*k*_*c*_) > 1. Chain-reaction bankruptcies include the following characteristics:

1. First bankruptcy firms tend to be larger than their chain-reaction bankruptcy firms, as shown in red in Fig 3(a).
2. The probability of chain-reaction bankruptcies between larger firms is higher than between smaller ones, as shown in red in Fig 3(b).

Result 1 suggests that smaller firms are likely not to bear the propagating risks when the first bankruptcy firm is larger. These results imply that a large firm’s bankruptcy is expected to spread a higher amount of bad debt than small cases. We note the functional form of *A*(*k*_*f*_, *k*_*c*_) from Fig 3(c) as follows;

$$\begin{array}{c} A\left(k_{f},k_{c}\right)\propto k_{f}^{\alpha }k_{c}^{\beta } \end{array}$$ (1)

Here, (*α*, *β*) ≃ (0.7 ± 0.1, −0.3 ± 0.1). Each *α* and *β* of the interaction kernel is positive and negative, respectively, meaning that the probability of cascading failure is higher between larger first bankruptcy firms and smaller trading firms.

## Results and discussion

### Monte Carlo simulation

**Fig 4:**
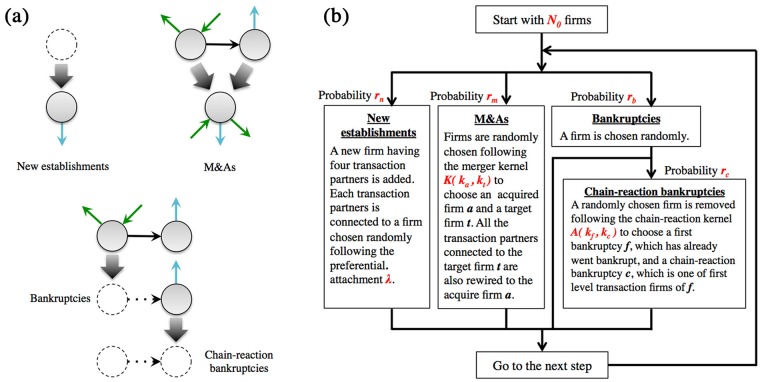
**(a)** The three basic processes for firms: new establishments, M&As, bankruptcies, and chain-reaction bankruptcies in our simulation model. **(b)** Time step flow chart of our simulation model. Each parameter set corresponds to the state of real business firm ecosystems in Japan [47]; the occurrence probabilities *r*_*n*_: *r*_*m*_: *r*_*b*_: *r*_*c*_ = 0.5: 0.15: 0.33: 0.02, the preferential attachment exponent for new comers λ = 1.0, and the merger kernel’s exponents *α*′ = 1.1, *β*′ = 0.7.

**Fig 5:**
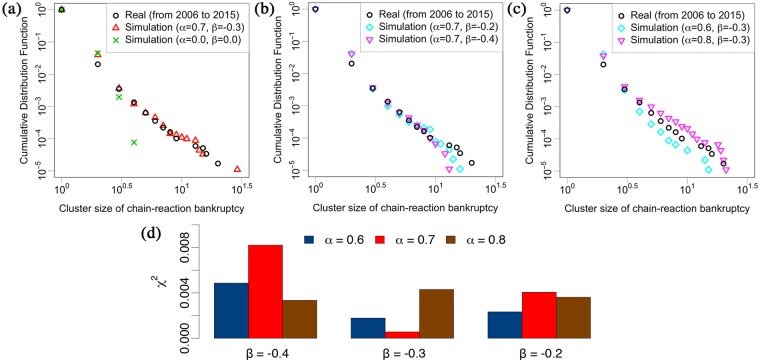
Cluster size distribution of chain-reaction bankruptcies in log-log plot. Black circles show the real distribution from 2006 to 2015. **(a)** Red triangles and green x-marks show the Monte Carlo simulation results with *α* = 0.7, *β* = −0.3 and *α* = *β* = 0.0 by 10 attempts, respectively. **(b,c)** Light blue squares and pink downward triangles show the **(b)** *α* = 0.7, *β* = −0.2 and *α* = 0.7, *β* = −0.4, and **(c)** *α* = 0.6, *β* = −0.3 and *α* = 0.8, *β* = −0.3 by 10 attempts, respectively. **(d)** Two-sample Kolmogorov-Smirnov statistical distribution (*χ*^2^) between real and simulated distribution. The smaller the *χ*^2^, the smaller the difference between real and simulated distribution. The cases that take into account of the interaction kernel of chain-reaction bankruptcies (red triangles) fit better with the real data even though the case that ignores the interaction kernel of chain-reaction bankruptcies (green x-marks) is similar to the previous study.

**Fig 6:**
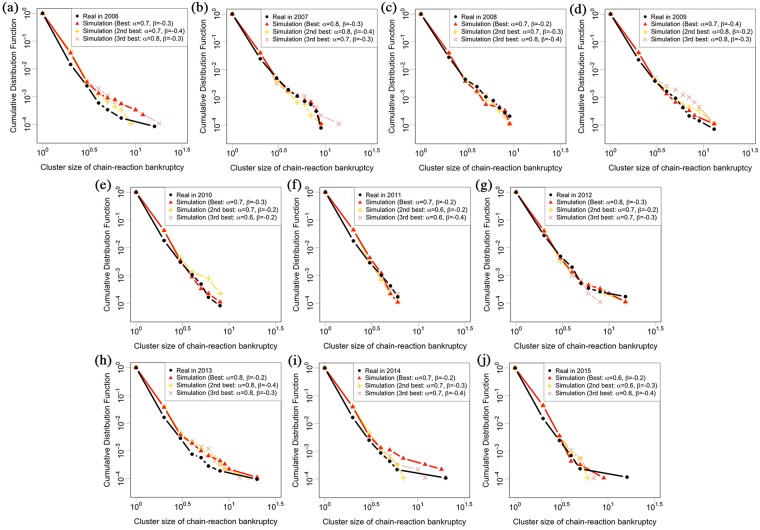
Cluster size distribution of chain-reaction bankruptcies in log-log plot. Black circles show the real distribution for each year. Red triangles, orange crosses, and pink x-marks show the Monte Carlo simulation results of the best parameters set, the second best parameters set, and the third best parameters set by judging from the median of *χ*^2^ of each parameters set by 20 attempts, respectively. **(a)** *α* = 0.7, *β* = −0.3 in 2006. **(b)** *α* = 0.8, *β* = −0.3 in 2007. **(c)** *α* = 0.7, *β* = −0.2 in 2008. **(d)** *α* = 0.7, *β* = −0.4 in 2009. **(e)** *α* = 0.7, *β* = −0.3 in 2010. **(f)** *α* = 0.7, *β* = −0.2 in 2011. **(g)** *α* = 0.8, *β* = −0.3 in 2012. **(h)** *α* = 0.8, *β* = −0.2 in 2013. **(i)** *α* = 0.7, *β* = −0.2 in 2014. **(j)** *α* = 0.6, *β* = −0.2 in 2015. Each *α* and *β* of the interaction kernel is positive and negative, respectively, without time variant despite the fact that the number of bankruptcies in Japan has gradually decreased since 2008 in Table 1.

It is expected that the empirically observed interaction kernel plays the role of the complexity underlying the firm ecosystem from the viewpoint of chain-reaction bankruptcies. In this section, we apply Eq 1 $A\left(k_{f},k_{c}\right)\propto k_{f}^{\alpha }k_{c}^{\beta }$ for numerical simulation and confirm whether the kernel can work for the reproduction of the cluster size distribution of chain-reaction bankruptcies as observed in Fig 1(e). Miura et al.[46] proposed a simple business network model in which money flow direction between a pair of firms is represented by a directed link connecting nodes, and they take into account the effects of new establishments, bankruptcies, and mergers and acquisitions (M&As) by creation of new nodes, removal of nodes, and aggregation of nodes together with links, respectively, as schematically shown in Fig 4(a). The model accurately reproduces basic statistical characteristics such as degree distribution of business network transactions. Additionally, by using a merger kernel estimated through an M&A data analysis, the model reproduces business network characteristics with the parameter set estimated by real firm data [47]. Here, we revise the model introducing a new effect of chain-reaction bankruptcy based on the empirically estimated kernel *A*(*k*_*f*_, *k*_*c*_) as follows;

**Step1** Start with *N*_0_ firms with real links given from the data of business transactions as shown in Table 1 (in Fig 5, *N*_0_ = 1,286,379 using the network data from 2009; in Fig 6, *N*_0_ depends on the year).
**Step2** Choose one of the following three events stochastically. The occurrence probabilities of new establishments, M&As, bankruptcies, and chain-reaction bankruptcies are denoted by *r*_*n*_, *r*_*m*_, *r*_*b*_, and *r*_*c*_, respectively (*r*_*n*_: *r*_*m*_: *r*_*b*_: *r*_*c*_ = 0.5: 0.15: 0.33: 0.02, which corresponds to the state of real business firm ecosystems in Japan [47]. In addition, *r*_*b*_ and *r*_*c*_ is estimated in Table 1).
  **New establishments** A new firm having four transaction partners (two in-links and two out-links) is added; it is roughly consistent with the rate between the number of firms and the number of transaction partners [47]. In addition, each transaction partner is connected to a firm chosen randomly following the preferential attachment rule with exponent λ (λ = 1.0 corresponding to the state of real business firm ecosystems in Japan [46][47]).
  **M&As** A pair of firms are randomly chosen following the merger kernel $K\left(k_{a},k_{t}\right)\propto k_{a}^{\alpha^{\prime }}k_{t}^{\beta^{\prime }}$ to choose an acquirer firm *a* and a target firm *t*. All transaction partners connected to the target firm *t* are also rewired to the acquirer firm *a* (*α*′ = 1.1, *β*′ = 0.7 corresponding to the state of real business firm ecosystems in Japan [47]).
  **Bankruptcies** A randomly chosen firm is removed along with all transaction links connected to this firm. This is because a firm’s lifetime follows an exponential distribution; it is roughly consistent with the simple assumption that a firm disappears randomly following a Poisson process [46, 47].
    **Chain-reaction Bankruptcies** A randomly chosen firm is removed, along with all transaction partners connected to this firm, following the interaction kernel of chain-reaction bankruptcies $A\left(k_{f},k_{c}\right)\propto k_{f}^{\alpha }k_{c}^{\beta }$ to choose a first bankrupted *f*, which has already gone bankrupt, and chain-reaction bankruptcies *c*, which are directly connecting the first bankrupt firm.
**Step3** Repeat **Step2** for *T* times (*T* = 25,000 in Figs 5 and 6, which corresponds to a year in accordance with average annual number of bankruptcy firms in Japan (Table 1) and it is also the typical time delay of failure cascade as already shown in Fig 1(f).

We illustrate our algorithm by a flow chart in Fig 4(b). Fig 5 show the analysis results of this numerical simulation with varying parameters. Black circles show the real distribution of chain-reaction bankruptcies accumulated from 2006 to 2015, which are shown in Fig 1(e). The parameters are **(a)** *α* = 0.7, *β* = −0.3 (red triangles), *α* = 0.0, *β* = 0.0 (green x-marks), **(b)** *α* = 0.7, *β* = −0.2 (light blue squares), *α* = 0.7, *β* = −0.4 (pink downward triangles), and **(c)** *α* = 0.6, *β* = −0.3 (light blue squares), *α* = 0.8, *β* = −0.3 (pink downward triangles), respectively. The simulation result that ignores the interaction kernel of chain-reaction bankruptcies (green x-marks) is similar to the previous study [39]; it clearly decays more quickly than the real cluster size distribution. On the other hand, the cases that take into account the interaction kernel of chain-reaction bankruptcy fit better with the real data. To find the best set of parameters of *α* and *β*, we apply the two-sample Kolmogorov—Smirnov test [48], which measures the difference between two distributions. The definition of the test statistic *χ*^2^ is $4D^{2}\frac{n_{1}*n_{2}}{n_{1}+n_{2}}$, where *D* is a maximum vertical deviation between two distributions and *n*_1_ and *n*_2_ are the number of samples of those distributions. From Fig 5(d), we find that the simulated distribution comes closest to the real one with the parameter set *α* = 0.7 and *β* = −0.3, the case of red triangles in Fig 5(a).

Fig 6 show the numerical analysis results of this simulation with different years to check the time dependency of the interaction kernel. The year and parameters are **(a)** *α* = 0.7, *β* = −0.3 in 2006, **(b)** *α* = 0.8, *β* = −0.3 in 2007, **(c)** *α* = 0.7, *β* = −0.2 in 2008, **(d)** *α* = 0.7, *β* = −0.4 in 2009, **(e)** *α* = 0.7, *β* = −0.3 in 2010, **(f)** *α* = 0.7, *β* = −0.2 in 2011, **(g)** *α* = 0.8, *β* = −0.3 in 2012, **(h)** *α* = 0.8, *β* = −0.2 in 2013, **(i)** *α* = 0.7, *β* = −0.2 in 2014, and **(j)** *α* = 0.6, *β* = −0.2 in 2015. Each parameter is estimated using the same method as in Fig 5. Black circles show the real distribution for each year. Red triangles, orange crosses, and pink x-marks show the Monte Carlo simulation results of the best parameters set, the second best parameters set, and the third best parameters set by judging from the median of *χ*^2^ of each parameters set by 20 attempts, respectively. It shows that *α* is positive and *β* is negative throughout the whole period.

## Conclusions

In this paper, we have shown the empirical and numerical analysis results of chain-reaction bankruptcies using a TDB Japanese firm database from 2006 to 2015. The main finding was that the kernel of chain-reaction bankruptcies was proportional to the product of *α*-th power of the size of first bankruptcy firms and *β*-th power of the size of chain-reaction bankruptcy firms similar to the case of money flow. We simultaneously found that *α* is positive and *β* is negative. It is obvious that the larger the first bankrupt firm and the smaller the trading firm, the higher the occurrence probability of cascading failure. Furthermore, we introduced simulations based on a network evolution model using inter-firm business transaction networks from 2006 to 2015 and showed that it reproduces the real cluster size distribution of chain-reaction bankruptcies. It shows that the interaction kernel is a key factor in expressing complexities of spreading bankruptcy risks on the real ecosystem. Moreover, we estimated that *α* is positive and *β* is negative throughout the whole period. This result suggests that the stability of firm ecosystems does not change from 2006 to 2015 despite the fact that the number of bankruptcies in Japan has gradually decreased since 2008.
